# Need satisfaction, motivational regulations and exercise: moderation and mediation effects

**DOI:** 10.1186/s12966-015-0226-0

**Published:** 2015-05-20

**Authors:** Karin Weman-Josefsson, Magnus Lindwall, Andreas Ivarsson

**Affiliations:** Research on Welfare, Health and Sport, Halmstad University, PO Box 823, SE 301 18 Halmstad, Sweden; Department of Psychology, University of Gothenburg, PO Box 500, SE 405 30 Gothenburg, Sweden; Department of Food and Nutrition, and Sport Science, University of Gothenburg, Box 100, SE 405 30 Gothenburg, Sweden; Centre for Person Centered Care, University of Gothenburg, Box 457, SE 405 30 Göteborg, Sweden

**Keywords:** Mediation, Moderation, Motivation, Self-determination

## Abstract

**Background:**

Based on the Self-determination theory process model, this study aimed to explore relationships between the latent constructs of psychological need satisfaction, autonomous motivation and exercise behaviour; the mediational role of autonomous motivation in the association of psychological need satisfaction with exercise behaviour; as well as gender and age differences in the aforementioned associations.

**Methods:**

Adult active members of an Internet-based exercise program (*n* = 1091) between 18 and 78 years of age completed a test battery on motivational aspects based on Self-determination theory. The Basic Psychological Needs in Exercise Scale and the Behavioural Regulation in Exercise Questionnaire-2 were used to measure need satisfaction and type of motivation and the Leisure Time Exercise Questionnaire to measure self-reported exercise.

**Results:**

Need satisfaction predicted autonomous motivation, which in turn predicted exercise, especially for women. Autonomous motivation was found to mediate the association between need satisfaction and exercise. Age and gender moderated several of the paths in the model linking need satisfaction with motivation and exercise.

**Conclusion:**

The results demonstrated gender and age differences in the proposed sequential mechanisms between autonomous motivation and exercise in the process model. This study thus highlights a potential value in considering moderating factors and the need to further examine the underlying mechanisms between needs, autonomous motivation, and exercise behaviour.

The positive effects of physical activity (PA) on health are well established, and it is generally accepted that regular PA and exercise can be used to prevent and treat a variety of physical and psychological diseases [see The European Health Reports: [1, 2] and to have beneficial effects on physical and psychological wellbeing [3]. A decade ago, Bull, Armstrong [4] stated that the effective promotion of a more physically active lifestyle has the potential to prevent as many as two million premature deaths and nearly 20 million disability-adjusted life years (DALYs) worldwide. Still, almost ten years later, health statistics show discouragingly low levels of PA and exercise levels (see [5]), and many interventions promoting PA and exercise are considered ineffective [6, 7]. Mounting literature strongly advocates the use of sound theory application in order to improve intervention efficacy (e.g., [8, 9]), and a lack of studies explaining the underlying processes (i.e., mechanisms) of theoretically derived hypotheses may partially account for many intervention failures [7]. In order to design successful interventions, an understanding of mediation models is fundamental for comprehending the complex interactions between theoretical constructs (e.g., motivation) and behaviour [7]. A widely used theory in modern research on human motivation is self-determination theory (SDT; [10, 11]), a framework that has received substantial support for its usefulness in health behaviour change (e.g., [12]) and in understanding exercise and PA behaviour (e.g., [13, 14]), as well as regarding proposed mechanisms of health behaviour change [15, 16].

Self-determination theory is a multidimensional theory based on the importance of people being self-determined in order to be motivated and engaged in certain behaviours [11]. A fundamental notion in SDT is that different types of motivation differ qualitatively along a continuum, in relation to the degree of self-determination or the extent to which the behaviour is regulated by controlling aspects. These relationships are described in a sub-theory called organismic integration theory (OIT; [17]). Amotivation represents one end of the continuum and is a lack of any intention to engage in the behaviour. At the other end of the continuum lies intrinsic motivation, the most self-determined, or autonomous, form of motivation. When intrinsically motivated, a person performs the behaviour volitionally because it feels inherently interesting or enjoyable. Extrinsic motivation, situated between amotivation and intrinsic motivation on the continuum, instead regulates the behaviour in order to achieve outcomes separate from the behaviour itself. There are four types of extrinsic motivation (external, introjected, identified and integrated regulation), which denote progressively more self-determined motives in relation to the degree to which the behaviour has been internalized. Internalization is a process where people integrate (social) values and behaviours into the self [17] and in this manner extrinsic motivation can become more self-determined (autonomous) when the behaviour is performed in order to achieve internalized outcomes [13]. As an example, integrated regulation signify a high level of internalization (social regulations fully integrated to the self), while introjection signify only partially internalized regulations [17]. With increased internalization, the motivation becomes more self-determined, which enhances persistence and adherence and internalization is of particular importance in maintaining non-enjoyable behaviours like exercise [11].

Another sub-theory of SDT, the basic needs theory (BNT), holds that self-determined motivation originates from individuals’ innate tendency to satisfy three basic psychological needs: competence, relatedness, and autonomy [17]. The need for competence denotes the feeling of effectance and capability, while autonomy represents feelings of volition or self-determination and relatedness refers to feelings of social inclusion and closeness. Ryan and Deci [17] define these basic psychological needs as “*innate psychological nutriments that are essential for on-going psychological growth, integrity and well-being*” (p 229) and suggest they are essential for understanding the content (what) and process (why) of human goals and behaviours. According to SDT [17, 18] and extensions of the SDT model [18, 19], self-determined motivation will be promoted when the three needs are satisfied. Also, it is proposed that self-determined motivation will lead to important behavioural, affective and cognitive outcomes, while the consequences are decreasingly positive from introjected and external motivation to amotivation [11]. A common trend in previous work is the strong inter-correlations between the three needs; and between competence and autonomy in particular (e.g., [20]), suggesting that the three needs may be captured by an underlying unidimensional factor. Supporting this view, Hagger, Chatzisarantis [21] found that a single global need satisfaction factor could explain latent variables representing autonomy, competence and relatedness.

A proposed key assumption [22] is that self-determined motivation mediates the association between need satisfaction and behavioural outcomes, also illustrated by the SDT process model proposed by [23]. Specifically linked to PA and exercise contexts, the model posits that a higher degree of satisfaction of needs (related to the behaviour) is suggested to be associated with increased exercise through a more self-determined motivation [22]. This relationship is supported by a considerable amount of research [13–15], also longitudinally in experimental designs [24], but the literature on how this link between needs, self-determined motivation and behavioural outcomes such as PA and exercise actually operates is somewhat inconsistent. Some studies [25] have found that need satisfaction and/or self-determined motivation are related to PA/exercise behaviour, whereas others (e.g., [26]) have found that self-determined motivation is unrelated to these behaviours. One reason for these different results could be the influence of various moderating factors (e.g., gender and age) on the associations between needs, motivation and behaviour. In addition, although several studies have supported the different individual paths of this proposed mediating model, few have fully tested the key assumption that self-determined motivation will mediate the association between need satisfaction and outcomes in the context of PA and exercise using recommended analyses also considering moderating effects. Edmunds and Ntoumanis [25], for example, found that the relationship between the need for competence and strenuous exercise was partially mediated by self-determined motivation (identified regulation). A limitation in their study, though, is that they used the simpler analytical regression strategy of Baron and Kenny [27], a method not recommended in modern research (see [28, 29], for example due to its conservative nature and low power [8]. McDonough and Crocker [26] used structural equation modelling (SEM), testing the mediation hypothesis, and found that the satisfaction of all three needs was related to self-determined exercise motivation but that self-determined motivation only partially mediated the effect on positive and negative affect and was unrelated to the behavioural outcome. The SEM analyses enable examination of measurement-free associations between constructs and more robust mediational paths.

In addition, neither of these two previous studies examined possible moderating factors such as gender or age, or the relationships between needs, motivation and outcomes, which may explain when and for whom need satisfaction and self-determined motivation may be related to outcomes and when and for whom self-determined motivation may mediate the proposed effect of need satisfaction on outcomes. This line of research questions has been raised as an important issue for future research [28, 29]. In a recent review on SDT in exercise and PA [14], more sophisticated analyses were specifically requested in order to clarify the role of need satisfaction in the development of self-determined motivation and to study possible moderating factors like gender and age differences. Following these recommendations, and considering that both age and gender could reasonably play an important role in the motivational processes within the SDT context, these moderating factors should be taken into account when studying these processes. Although basic psychological needs are thought to be universal and apply across genders, ages and cultures, it is likely that such factors could influence the means by which basic needs are met [17], how well the social context support need fulfilment, as well as how behavioural regulations emerge. Over the course of a lifetime, people’s reasons for engaging in exercise and PA may change along with aspects like natural variations in things like values, health and goals [30]. For example, older adults tend to have more intrinsically oriented exercise goals and motives [31], suggesting that they may have a more autonomous exercise motivation. Research findings regarding gender differences are mixed. Some studies imply that women have a general tendency towards more controlled regulations (mainly introjected) than men [14] while some suggest the opposite, that women are more autonomously regulated and men more externally regulated to exercise behaviour [32]. At the same time, a meta-analysis found only negligible gender differences in motivational regulations [33]. These mean-level results suggest that there is reason to also expect possible moderators in the pathways between need satisfaction, regulation and outcomes (such as gender and age differences). The identification of such moderators would provide important information for the theoretical understanding of SDT-based models of exercise [14].

The main aim of this study was to explore: (a) theoretically derived hypotheses about the relationships between the latent (free of measurement error) constructs of psychological need satisfaction, autonomous motivation, and the manifest variable of exercise behaviour; (b) the mediational role of autonomous motivation in the association of psychological needs with exercise behaviour; and (c) gender and age differences (moderating effects of gender and age groups) in the aforementioned associations.

## Methods

### Participants

The participants (*N* = 1091)–286 men and 805 women, aged 18-78 years (*M* = 45.0; *SD* = 11.7)–were all active Swedish members of an Internet-based exercise programme created by a Swedish company in the e-health industry offering web-based health-care services (e.g., pedometer step contests, weight-loss programmes, etc.) mainly in the private sector. Hence, the sample was expected to be diverse regarding, for instance, fitness level, age and gender, as well as motivational aspects.

### Measures

The Basic Psychological Needs in Exercise Scale (BPNES; [34]) measures satisfaction of the three needs autonomy, competence and relatedness in the exercise domain through 12 items (e.g., *“The way I exercise is in agreement with my choices and interests”*) and a five-point Likert scale, where 1 = “*I don’t agree at all*” and 5 = “*I completely agree*”. Cronbach’s alpha for the BPNES was 0.81 to 0.92 in the present study. The BPNES has been successfully validated as supporting the theoretically based three-factor model and the needs hypothesis of SDT [34]. It has also demonstrated gender invariance [35]. Motivation quality was measured by the Behavioural Regulation in Exercise Questionnaire-2 (BREQ-2; [36]) which has 19 items (e.g., *“It’s important to me to exercise regularly”*) and a five-point Likert scale, where 0 = “*not true for me*” and 4 = “*very true for me*”. The present study applied a four-pointed Likert with the same anchors, i.e., 1 = “*not true for me*” and 4 = “*very true for me*” scale. The scale measures behavioural regulations through five factors: extrinsic, introjected, identified and intrinsic motivation, and, unlike the original BREQ, the BREQ-2 also measures amotivation. In addition to using the five different regulations in BREQ-2 we followed the example of Sebire, Standage [37], and created one controlled motivation factor (external regulation and introjected regulation) and one autonomous motivation (identified regulation and intrinsic motivation) factor. Cronbach’s alpha for the BREQ-2 factors were 0.73 to 0.86 in the present study. The Leisure Time Exercise Questionnaire [38] was used to measure self-reported exercise. The LTEQ consists of three questions regarding the frequency of performing strenuous, moderate and light exercise during a regular week. The total exercise score can be calculated and transformed into the metabolic equivalent of exercise (MET) scores.

The BPNES and BREQ-2 were translated from English into Swedish according to the back-translation method [39]. A bilingual (English and Swedish) expert first translated the tests from English into Swedish, and then another bilingual expert translated them back into English. Differences in the translated versions and the originals were discussed in the research group and formed the foundation of the final versions. For the invariance and moderation analyses, mean age (45.0) was used to create two age groups: a younger (18-45 years) and an older (46-78 years) one.

When testing the factor validity and invariance of the translated BPNES and BREQ-2 scales, the theoretical a priori models displayed adequate-to-good fit with data. For the BPNES, the theoretical a priori three-factor model demonstrated good fit with data: Satorra-Bentler *χ*2 = 246.45 (51df), *CFI* = 0.96; *RMSEA* = 0.059 (0.052-0.067). The five-factor model of the BREQ-2 demonstrated acceptable fit to data: *χ*2 = 408.60 (142df), *CFI* = 0.94; *RMSEA* = 0.044 (0.039-0.049). All standardized factor loadings were significant and generally over .60. The three-factor measurement model of the BPNES displayed strict invariance (i.e., invariant residuals in addition to invariant factor loadings and intercepts) across gender and age, as the CFI did not decrease more than .01 in model fit when factor loadings, intercepts, and residuals were constrained to be equal across groups of gender (men and women) and different ages (18–45 years, and 46–78 years). The BREQ-2 measurement model demonstrated strong invariance (i.e., invariant factor loadings and intercepts) across gender and across age groups.

### Procedures

The study began with a pilot study including ten persons selected through convenience sampling to test the comprehension and design of the test battery, using the think-aloud method [40]. The pilot study resulted in the clarification and remodelling of parts of the test battery for the final version. Following a list of members provided by the e-health service company, potential participants for Study 1 were contacted by e-mail, with information on the aim of the study, ethical concerns and practical issues. When logging in to the questionnaire, the participants had to tick a box for informed consent in order to access the questionnaire The collected data were stored in a certain web account accessible only by the researchers. Participation was anonymous, and no personal data were requested; hence, no personal register was created. The study was approved by the regional ethical board.

### Analysis

*T*-tests were conducted to examine differences in psychological need and motivation across gender and age-groups., In the main analyses, structural equation modelling (SEM) and mediation analysis using a bootstrapping resampling approach [28, 41] were used, enabling the examination of measurement-free associations between constructs and more robust mediational paths. *Mplus* version 7.1 (Muthen & Muthen, 1998–2009) was used to analyse the data with the robust maximum likelihood (MLR) estimators. Missing data were handled using a full maximum likelihood (FIML) estimator, which is default in Mplus. Therefore, data from all (*N* = 1091) participants (i.e., including those who had missing data on some items or variables) were used in the *Mplus* analyses. Based on recommendations by Hu and Bentler [42], the following fit indexes were used: (a) Satorra-Bentler chi-square statistics, (b) Bentler’s comparative fit index (*CFI*; [43]), and (c) the root mean square error of approximation (RMSEA; [44]). For the *CFI*, values close to or greater than 0.95 indicate a well-fitting model [42]. For the *RMSEA*, values less than .05 indicate a good fit, whereas values up to .08 represent a reasonable fit [44]. In the invariance testing, we used the recommendations by Cheung and Rensvold [45]. Because the chi-square difference test is sensitive to the sample size, they recommend using a decline in the *CFI* of 0.01 or less as indicative of invariance. Moderation analyses were conducted through multi-group analyses, whereby model fit for models with no constraints between groups (e.g., men vs. women) in terms of paths was compared with model fit in models in which certain paths were constrained to be equal between groups.

**Fig. 1:**
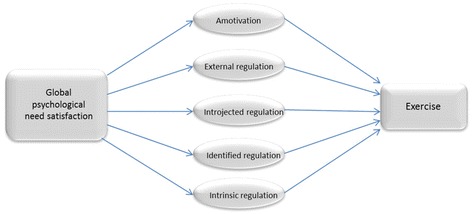
The structural equation model used to examine the relations between psychological need satisfaction, motivational regulations, and exercise

Mediator models with a bootstrapping resampling approach for calculating product-of-coefficients and asymmetric 95 % confidence intervals based on 1000 resamples [29, 41] were used to test indirect effects. All mediation analyses were performed in Mplus, using latent variables for the need satisfaction (BPNES) and motivation (BREQ-2) variables. Moreover, bias-corrected and accelerated bootstrap confidence intervals for the indirect effects were used [29]. Bootstrap confidence intervals are recommended because they do not make unrealistic assumptions about the shape of the sampling distribution of the indirect effect like, for example, the Sobel test does [29]. In the analyses, indirect effects of the independent variable (in the present study, psychological need satisfaction) on the outcome variable (exercise) through the proposed mediator variables (e.g., autonomous motivation) were estimated (see Fig. 1). The Wald Chi-Square test in Mplus was used to test if gender or age moderated the mediational effects of motivation.

## Results

Descriptive data divided into gender and age groups are presented in Table 1. The *t*-tests revealed men to report higher external regulation (*t* (1054) = 2.04, *p* < .05), higher introjected regulation (*t* (1046) = 2.11, *p* < .05) and more strenuous exercise (*t* (1010) = 2.52, *p* < .05), but less moderate exercise (*t* (1012) = −3.95, *p* < .05) compared to women. Turning to age differences, younger adults reported higher external (*t* (1018) = 2.35, *p* < .05) and introjected (*t* (1000) = 5.93, *p* < .01) regulation, and were also engaged in more strenuous (*t* (968) = 4.9, *p* < .01) and total exercise (*t* (966) = 2.56, *p* < .01) than older adults, who instead were more engaged in moderate exercise (*t* (966) = 2.56, *p* < .01) than younger adults.

**Table 1:** Descriptives (means and standard deviations) of psychological need satisfaction (BPNES), behavioural regulations (BREQ-2), and exercise (LTEQ) last six months (PA), across gender and age groups

	Gender		Age		
	Men	Women	Younger	Older	Total
	(*n* = 286)	(*n* = 805)	(18–45)	(46–78)	sample
			(*n* = 539)	(*n* = 501)	(N = 1091)
BPNES					
Autonomy	4.0 (.84)	4.1 (.80)	4.0 (.81)	4.1 (.81)	4.1 (.81)
Competence	3.8 (.84)	3.8 (.82)	3.8 (.82)	3.8 (.83)	3.7 (.82)
Relatedness	3.9 (1.1)	3.8 (1.0)	3.8 (1.00)	3.8 (1.09)	3.8 (1.0)
Global need	11.7 (2.3)	11.7 (2.3)	11.6 (2.2)	11.7 (2.3)	11.7 (2.3)
BREQ-2					
Amotivation	1.1 (.30)	1.1 (.24)	1.1 (.28)	1.1 (.23)	1.1 (.25)
External reg.	1.2 (.39)	1.1 (.34)	1.2 (.38)	1.1 (.32)	1.1 (.35)
Introjected reg.	2.1 (.73)	2.2 (.78)	2.3 (.75)	2.0 (.77)	2.2 (.77)
Identified reg.	3.2 (.66)	3.2 (.63)	3.2 (.65)	3.2 (.62)	3.2 (.63)
Intrinsic reg.	3.2 (.70)	3.3 (.69)	3.3 (.70)	3.3 (.67)	3.3 (.70)
LTEQ					
Strenuous	2.2 (1.8)	1.8 (1.6)	2.1 (1.9)	1.6 (1.5)	1.9 (1.7)
Moderate	2.9 (3.5)	3.7 (2.6)	3.3 (2.4)	3.7 (3.3)	3.5 (2.9)
Light	3.6 (4.1)	3.8 (2.9)	3.8 (3.2)	3.6 (3.3)	3.7 (3.3)

global need = total need satisfaction factor; reg = regulation

### Psychological need satisfaction and controlled and autonomous motivation predicting exercise

When using the three need satisfaction factors simultaneously to predict motivation in the analyses, we found that whereas competence and relatedness predicted autonomous motivation in expected positive directions (*β* = .89, *p* < .01 and *β* = .15, *p* < .01 respectively) the path between autonomous need satisfaction and autonomous motivation was negative and significant (*β* = −.33, *p* < .01), which was unexpected. Given that the correlations between autonomy and the latent factors of the BREQ-2 were according to expectations (i.e., positive correlations with identified and intrinsic regulation but negative correlations with amotivation and external regulation), the negative path displayed in the model between autonomy and autonomous motivation most probably signals a suppressor effect rather than a conceptually meaningful result. Because the latent factors of competence, autonomy and relatedness correlated moderately to strongly, the three psychological need satisfaction factors were collapsed into one total psychological need factor, using a second-order (higher-order) model.

In the first step, a model including the total psychological need satisfaction factor predicting autonomous and controlled motivation, which in turn predicted exercise behaviour (METS), was tested. The autonomous factor consisted of the BREQ-2 items hypothesized to load on the identified regulation and intrinsic motivation factors whereas the controlled motivation factor were comprised of the items hypothesized to load on the external regulation and introjected regulation factors. Collapsing items this way to create the autonomous and controlled motivation factors resulted in a suboptimal fit according to the CFI, but reasonable fit according to the RMSEA for the full group: Satorra-Bentler *χ*2 (345) = 2019.90, *p* < .001; *CFI* = .86; RMSEA = .067 (90 % *CI* = .064 to .070). The results from the model are presented in Table 2. The path from total psychological need satisfaction to controlled motivation was weakly negative and significant (*β* = −.26, *p* < .01), whereas the path from need satisfaction to autonomous motivation was strongly positive and significant (*β* = .68, *p* < .01).

**Table 2:** Predictions between psychological need satisfaction, motivational type (controlled vs. autonomous motivation) and exercise across gender and age groups (standardized estimates)

Regression weights	Men	Women	Younger	Older	Full sample
			(18–45)	(46–78)	
	*n* = 286	*n* = 805	*n* = 539	*n* = 550	*N* = 1091
Need → Contmot	-.20*	-.29**	-.21**	-.30**	-.26**
Need → Autmot	.72**	.66**	.66**	.70**	.68**
Contmot → Exercise	.26**	-.07	-.03	.08	.02
Autmot → Exercise	.23*	.35**	.35**	.31**	.33**

Need = total need satisfaction factor; Contmot = Controlled motivation (combined external and introjected regulation); Autmot = Autonomous motivation (combined identified regulation and intrinsic motivation) *p < .05; **p < .01. A complete table with standard errors for the estimates can be obtained by request from the first author

In the next step, the two factors controlled and autonomous motivation were replaced with the five factors of the BREQ-2, to offer more specific insight into how various types of motivation regulation were associated with total need satisfaction and exercise behaviour. This model is illustrated in Fig. 1. For the full sample (see Table 3, last column to the right), total need satisfaction, as modelled by the higher-order factor, significantly and inversely predicted amotivation (*β* = −.44, *p* < .01) and external regulation (*β* = −.26, *p* > .01) but not introjected regulation. Moreover, total need satisfaction predicted both identified regulation (*β* = .79, *p* < .01) and intrinsic motivation (*β* = .81, *p* < .01). In terms of the paths from type of motivation to exercise, only identified regulation significantly (*β* = .30, *p* < .01) predicted exercise behaviour for the full sample.

**Table 3:** Psychological need satisfaction and self-determined motivation (All five BREQ-2 factors) predicting exercise across gender and age groups (standardized estimates)

	Men	Women	Younger (18-45)	Older (46–78)	Full sample
	*n* = 286	*n* = 805	*n* = 539	*n* = 501	*N* = 1091
Need → Amot.	-.44 (.07)**	-.44 (.04)**	-.46 (.05)**	-.43 (.05)**	-.44 (.06)*
Need → Ext. reg.	-.14 (.07)*	-.30 (.04)**	-.24 (.05)**	-.29 (.05)**	-.26 (.04)*
Need → Introj. reg.	.41 (.07)**	-.03 (.05)	.11 (.06)*	.08 (.06)	.09 (.06)
Need → Ident. reg.	.88 (.04)**	.75 (.03)**	.78 (.03)**	.81 (.03)**	.79 (.06)*
Need → Intr. mot.	.84 (.03)**	.79 (.02)**	.76 (.03)**	.86 (.02)**	.81 (.06)*
Amot. → Exercise	-.10 (.09)	.00 (.05)	-.02 (.06)	-.02 (.07)	-.02 (.04)
Ext. reg. → Exercise	.26 (.08)**	-.00 (.04)	.04 (.05)	.12 (.06)*	.07 (.05)
Introj. reg. → Exercise	.12 (.10)	-.14 (.05)*	-.15 (.06)**	-.06 (.06)	-.08 (.05)
Ident. reg. → Exercise	-.06 (.16)	.40 (.07)**	.52 (.08)**	.11 (.11)	.30 (.11)*
Intr. mot. → Exercise	.16 (.14)	.04 (.07)	-.06 (.08)	.24 (.10)*	.08 (.06)

Need = total need satisfaction factor; Amot. = Amotivation; Ext. reg. = External regulation; Introj. reg. = Introjected regulation; Ident. reg. = Identified regulation; Intr. mot. = Intrinsic motivation. * *p* < .05; **p < .01. A complete table with standard errors for the estimates can be obtained by request from the first author

We also examined the relationships of different exercise intensities with psychological need satisfaction and motivation regulation, modelled as latent constructs. The results are described in Table 4. In general, the correlations between self-reported light exercise intensity with needs and regulations were very small and non-significant. Moderate exercise intensity was weakly but significantly associated with the needs autonomy (Φ = .17, *p* < .01) and competence (Φ = .19, *p* < .01) and with amotivation (Φ = −.11, p < .01), identified regulation (Φ = .11, *p* < .01) and intrinsic motivation (Φ = .14, *p* < .01). Global need was weakly correlated with moderate exercise (Φ = −.18, p < .05), but moderately correlated with strenuous (Φ = .43, *p* < .01) and total (Φ = .35, *p* < .01) exercise. Strenuous exercise intensity demonstrated the most robust pattern of relationships with needs and regulations with significant associations with all three needs and all five regulations. The correlations with the three needs and identified regulation and intrinsic motivation were moderate in size (Φ = .47-.31, *p* < .01), whereas the correlation with amotivation (Φ = −.17, p < .01) and extrinsic regulation (Φ = −.11, p < .01) were weak and negative. Mirroring how the total exercise score of METS is calculated (putting heavy focus primarily on frequency of strenuous exercise), the correlations of total exercise score with needs and regulations were slightly lower than for strenuous exercise, but higher than for moderate and light exercise.

**Table 4:** Correlations between different exercise intensities and latent variables of psychological needs and motivational regulations

	Exercise intensity			
	Light	Moderate	Strenuous	METS
BPNES				
Autonomy	-.02	.17*	.33**	.31**
Competence	-.07	.19*	.46**	.36**
Relatedness	-.07	.04	.31**	.17*
Global Need	-.06	.18*	.43**	.35**
BREQ-2				
Amotivation	.05	-.11*	-.17*	-.16*
Extrinsic	.05	.01	-.11*	-.03
Introjected	-.02	-.02	.12*	.03
Identified	-.05	.11*	.47**	.33**
Intrinsic	-.04	.14*	.36**	.29**

METS = metabolic equivalent of exercise, a total weighted exercise score; global need = total need satisfaction factor; *p < .05; **p < .01

### Moderating effects of age and gender

The moderating effects analyses were conducted using the total exercise score. Only one significant difference across age groups or gender was found in the model including controlled and autonomous (see Table 3). Controlled motivation positively predicted exercise for men (*β* = .26, *p* < .01) but not for women (*β* = −.07, *p* = .07), mirrored by a significant decrement in model fit (Δ *χ*^2^ = 13.78/ 1 *df*) when these paths were constrained to be equal across men and women.

When including the five separate BREQ-2 factors in the model, a number of significant differences in strength and direction of paths between men and women appeared (see Table 4). Model fit decreased significantly (reflecting a significant difference between men and women) when the following paths were constrained to be equal: total need satisfaction to introjected regulation (Δ *χ*^2^ = 24.06/ 1 *df*); total need satisfaction to identified regulation (Δ *χ*^2^ = 4.09/ 1 *df*); external regulation to exercise (Δ *χ*^2^ = 9.84/ 1 df); introjected regulation to exercise (Δ *χ*^2^ = 11.67/ 1 *df*); and identified regulation to exercise (Δ *χ*^2^ = 28.02/ 1 *df*). Looking more specifically into these differences, the path between total need satisfaction and introjected regulation was positive and significant for men (*β* = .41, *p* < .01) but negative and non-significant for women, and total need satisfaction was more strongly related to identified regulation for men (*β* = .88, *p* < .01) than for women (*β* = .75, *p* < .01). Moreover, external regulation predicted exercise for men in a positive direction (*β* = .26, *p* < .01) but was not related to exercise for women, and introjected regulation was positively but non-significantly associated with exercise for men but negatively and significantly associated with exercise for women (*β* = −.14, *p* < .05). Finally, identified regulation predicted exercise for women (*β* = .40, *p* < .01) but not for men.

When differences in paths between age groups were examined, significant differences were noted in the two paths: identified regulation to exercise (Δ *χ*^2^ = 7.19/1 df), where the path was stronger and significant for the younger year group (*β* = −.52 *p* <> .01) compared to the older one (*β* = .11, *p* > .05), and intrinsic motivation to exercise (Δ *χ*^2^ = 5.87/ 2 df), where the path was negative and non-significant (*β* = −.06, *p* > .05) for the young group but positive and significant (*β* = .24, *p* < .05) for the older one.

### The mediating effect of autonomous motivation in the association of psychological need satisfaction with exercise

The mediating (indirect) effects of controlled and autonomous motivation and the separate BREQ-2 factors are presented in Table 5. In the full sample there was a significant indirect effect of the autonomous motivation (*αβ* = 5.42, 95 % *CI* = 4.21-6.52), indicating that autonomous motivation acted as a mediating variable in the relationship between psychological need satisfaction and exercise. There was however no indirect effect of controlled motivation. Looking more specifically at the mediating effects of the different BREQ-2 factors, indirect effects were found for amotivation (*αβ* = 0.49, 95 % *CI* = 0.06-0.92), identified regulation (*αβ* = 2.70, 95 % *CI* = 1.05-4.10) and intrinsic motivation (*αβ* = 1.83, 95 % *CI* = 0.49-3.12).

**Table 5:** The mediating (indirect) effects of self-determined motivation in the relationship between psychological need satisfaction and exercise across gender and age group

Bootstrap results for indirect effects					
Indirect effects	Men *aβ* (95 % *CI*)	Women *aβ* (95 % *CI*)	Younger (18–45) *aβ* (95 % *CI*)	Older (46–78) *aβ* (95 % *CI*)	Full sample *aβ* (95 % *CI*)
Controlled motivation	−1.91* (−6.83–−0.37)	0.12 (−0.44–0.58)	−0.16 (−1.09–0.37)	−0.59 (−3.73–0.22)	0.29 (−0.44–1.03)
Autonomous motivation	4.30* (1.60–6.49)	5.60* (4.31–6.90)	7.28* (4.25–10.14)*	3.98* (2.30–5.66)	2.35* (0.99–3.71)
Amotivation	0.71* (0.10–2.27)	0.45 (−0.09–0.99)	0.71 (−0.29–1.81)	0.43 (−0.40–1.45)	0.49* (0.06–0.92)
External regulation	−2.27* (−7.60–−0.50)	0.16 (−0.28–0.57)	−0.13 (−1.07–0.44)	−0.49 (−2.89–0.26)	−0.39 (−1.40–0.11)
Introjected regulation	1.48* (0.35–4.26)	−0.10 (−0.53–0.26)	−0.10 (−1.00–0.20)	−0.05 (−0.91–0.18)	−0.09 (−0.38–0.02)
Identified regulation	2.95 (−5.94–7.69)	2.72* (1.57–3.93)	3.06 (−0.24–7.56)	2.57 (−0.41–4.79)	2.70* (1.05–4.10)
Intrinsic motivation	3.17* (0.88–6.05)	1.39 (−0.11–2.83)	−0.82 (−4.94–3.07)	1.12 (−0.38–2.75)	1.83* (0.49–3.12)

Note: *αβ =* product-of-coefficient estimate (95 % bias-corrected and accelerated bootstrap CI based on 1000 bootstrap resamples); Controlled motivation = external regulation + introjected regulation); Autonomous motivation = identified regulation + intrinsic motivation; * *p* < .05

As a consequence of the differences in paths between need, motivation and exercise for men and women, the indirect effects of motivation also differed, in particular for controlled motivation, external regulation and introjected regulation (see Table 5). For example, the indirect effect of controlled motivation was negative and statistically significant for men but positive and non-significant for women. Also, the indirect effect of external regulation was negative and significant for men but positive and non-significant for women and the indirect effect of introjected regulation was positive and statistically significant for men but negative and non-significant for women. However, gender or age did not significantly moderate any of the indirect effects as no Wald Chi-Square tests were significant.

## Discussion

The purpose of the present study was to examine key pathways in a self-determination based model of motivation in exercise, linking satisfaction of psychological needs with autonomous motivation and exercise, and specifically to look at how gender and age may moderate these pathways. Moving from first-generation research questions targeting whether relationships exist to second-generation research questions focusing on the conditions under which, and when, relationships exist and, finally, to third-generation questions targeting mechanisms of change (mediators) in relationships entails vital steps in the progress of knowledge development in any field [46]. SDT-related exercise research has typically evolved around first- but not second- or third-generation research questions, resulting in a gap in the knowledge base regarding what factors moderate and mediate key relationships in the theory. Our main analyses in the full sample revealed that higher need satisfaction predicted autonomous motivation, and that autonomous motivation in turn predicted behavioural outcomes in terms of more exercise. Thus, our results are well in line with general SDT stipulations and previous research (e.g., [13–15, 23, 24, 47]). Moreover, our study advanced previous work by demonstrating differences in paths between need satisfaction, motivation and exercise. For example autonomous motivation, and in particular identified regulation, was a stronger predictor of exercise for women compared with for men, whereas controlled motivation was positively associated with exercise in men but not in women. Overall these findings contribute with interesting information regarding how the theoretically hypothesized associations between need satisfaction, motivation and exercise may be moderated.

In essence, we found that autonomous motivation, but not controlled motivation, mediated the relationship between psychological need satisfaction and exercise. Thus, this study further confirms previous suggestions that the relationship between psychological need satisfaction and outcomes is mediated by motivation [22, 23, 47], adding to the understanding of the underlying mechanisms of SDT constructs and how they can influence behaviour. Because comparable previous studies have demonstrated only partial [25] or no mediating effects of self-determined motivation [26] using non-recommended mediation analysis [28, 29], our study seems to be one of the first to demonstrate the mediating effect of self-determined motivation in the relationship between need satisfaction and outcomes in the context of exercise, while also considering moderating effects.

Although the differences across gender and age in the mediation effects of motivation were not statistically significant, there were indications that the potential mechanisms through which need satisfaction influence exercise may differ in direction and strength across subgroups such as gender. In order to design effective exercise interventions, previous work has proposed that “one size may not fit all” [48] and that further investigation is needed concerning cross-study differences in SDT-related relationships regarding gender [33] as well as age and other potential moderating factors [14]. Most paths in the model were invariant according to SDT [11], but at some points we found pathway differences between age and gender groups, not only using controlled and autonomous motivation factors but also for the subscale regulations (identified and intrinsic regulations, as well as amotivation), which paints a slightly more complex picture of the mechanisms responsible for exercise behaviour. As stated by Hayes [49], *“…an analysis that ignores the potential contingencies and boundary conditions of an effect is going to result in a greater oversimplification of complex processes relative to an analysis that acknowledges that complexity by formally modelling it…”* (p. 327). In this way, our study represents a primary attempt to create a better understanding of the SDT process model in terms of behavioural outcome. Since the nuts and bolts of how this would inform intervention design more specifically still remain unclear, the results of this study highlight the need for further inquiry regarding possible moderating effects. We therefore agree with previous research that addressing age and gender issues could benefit practice [14] and propose future studies to more thoroughly examine notions of both mediating and moderating effects.

Offering simple explanations for the demonstrated moderating effects of age and gender is not easy, and due to the exploratory nature of the moderation analyses such clarifications would seem quite premature. Previous research mainly concerns mean-level observations, and does not contribute a rich frame of reference for explaining the specific differences in paths between men and women and different age groups (e.g., [14, 33]). Nevertheless, the choice to examine these paths is based on some preliminary thoughts that should be expanded on. For example, there is reason to believe that factors like age and gender could influence the means by which, for example, basic needs are satisfied [11, 17], and it is likely that people’s reasons for exercise change over their lifespans [30]. Although both identified regulation and intrinsic motivation are highly self-determined and integrated into the self, previous research has shown that older adults have more intrinsically oriented exercise goals and motives than younger adults do (e.g., [31, 50]). Accordingly, the current results (demonstrating that intrinsic regulation positively predicts exercise only for older adults, whereas identified regulation was a stronger exercise predictor among younger adults) might support these previous mean-level studies by indicating that the older adults in this sample were somewhat more autonomously regulated than the younger adults. Furthermore, these mediating effects might not be moderated by age per se, but perhaps age serves as a proxy in terms of different stages in life possibly offering different opportunities and barriers to choose between leisure time activities with various degrees of need support (or need thwarting). Such arguments imply that the moderated mediation effects found in this study merely represent a first step towards the exploration of possible underlying mechanisms and other potential mediators. Further investigation of moderated mediation effects is needed in order to offer deeper knowledge of how to address these mechanisms, perhaps by studying how the social contexts might differ for the different sub groups (men/women and younger/older adults) and thereby may or may not support need fulfilment related to barriers and opportunities in the social environment. It should be noted that when the sample was split into three age groups (younger, middle-aged and older adults) the findings on age differences remained essentially similar to those based on two age groups.

In regard to gender, the present results add to the inconsistency shown in previous studies on mean level by displaying more controlled (external and introjected) regulations predicting exercise for men, whereas more autonomous (identified) regulation predicted exercise for women, which is in line with the study by Li [32] but quite the opposite of the conclusions in the review by Teixeira, Carraca [14], and non-consistent with, for example, research by Guérin, Fortier [33]. These inconsistencies point to a clear need to further examine gender-related differences in how regulations are associated with exercise; and the arguments above regarding other potential underlying dimensions of age moderating the mediation effects also apply to the discussion of gender (e.g., social-environmental factors).

There is also reason to mention something about relations between the different exercise intensities, psychological needs and motivational regulations. Light exercise was not found to be related with needs or motivational regulations and moderate exercise was only weakly related to two needs and three regulations. Strenuous exercise, however, was shown to be moderately associated with all three needs, identified regulation and intrinsic motivation and also significantly but weakly associated with the other three motivation regulations. These results are in line with previous findings on exercise intensity and motivational regulations (e.g., [51, 52]). As has been forwarded by others [50], light and moderate exercise may incorporate activities such as walking and cycling that are more habitual in nature and therefore will be less affected by cognitive processing compared to more structured and strenuous types of exercise. Also, strenuous activities may require more engagement, planning and self-regulation skills than light and moderate activities. Future studies should further explore different predictors/models for the different intensities.

The strong competence-autonomy association in our study most probably resulted in the suppressor effect, which was demonstrated by an unexpected and, from a theoretical viewpoint illogical, negative association in the model between autonomy and autonomous motivation. The suppressor effect was handled in our study by collapsing the three needs into one global latent need satisfaction factor. This modified model fit data well, which is in line with the findings in previous work (e.g., [20, 21]) that a single global need satisfaction factor could explain latent variables representing autonomy, competence and relatedness. Moreover, these results are also in line with the assertion [17, 53] that the satisfaction of the three needs is complementary; that is, that the satisfaction of one need (e.g., autonomy) can occur only if the other needs are also satisfied.

### Limitations and future directions

The current results should be interpreted cautiously with regard to methodological differences in previous studies (i.e., traditional versus more advanced methods like SEM), and the somewhat contradictive results call for further inquiries concerning these moderators, using advanced analysis and various samples. The assumption of linear relations between motivational and behavioural variables should also be taken into consideration, since associations could be non-linear. Interpretation should also consider the specific sample. First, the sample consists of members of a web-based exercise programme focused on step contests and weight loss, which might have impacted participative motives, regulations and preferences; second, the relatively high mean age (45 years) combined with the female dominance could also have affected the results (e.g., women being more autonomous due to higher age); and third, this specific population of older adults might not be representative of this age group in general (e.g., these web-based exercise services might attract older adults with certain capacities and/or characteristics). Furthermore, women seem be more inclined to join web-based PA interventions (see e.g., [54–56]), which might serve as a complementary reason why women were more autonomously motivated in this study than men were. It is also possible that men are mainly attracted by the competitive features of the web service, which might be expected to pull for more controlled motivations per se. The primary limitation of the study, however, is the cross-sectional design. Therefore, even though proper analyses were used [8] also for cross-sectional data [57], and although a direction is implied in our analytical model (e.g., need satisfaction predicting motivation), we cannot rule out the possibility of reversed causation. Moreover, drawing conclusions about mediation analyses based on cross-sectional data may be misleading because mediation consists of processes that unfold over time [58]; future studies should therefore further examine whether the mediating role of autonomous motivation will hold longitudinally. Also, using the revised four-point Likert BREQ-2 scale instead of the original five-point scale may have resulted in less variation and higher total scores, primarily for amotivation and external regulation. Nevertheless, the analyses showed the factor structure to be stable also with the revised four-point scale.

Finally, even though a comparatively reliable [59] and valid [60] self-report measure of exercise was used, it is unquestionably less reliable than objective measures. On the other hand, we employed a large e-health based sample of non-clinical middle-aged adults, who are more rarely studied in the context of motivation and exercise. In addition, we conducted age- and gender-specific analyses, contributing new information in terms of when and for whom the different paths in the SDT model exist or are stronger/weaker, but more work is needed in order to understand how such mediating and moderating effects and mechanisms adds to the growing evidence of SDT utility [12–15] and potential practical implications. Making the best use of SDT as a kind of compass in constructing exercise interventions could assist a smoother and more sustainable transition from inactivity to activity for inactive individuals and our results highlight the importance for researchers in the future to examine the effect of potentially relevant moderating factors that may influence the different paths in the SDT-based models, rather than controlling these variables (e.g., age and gender), as typically tends to be done.

## Conclusions

Based on sophisticated mediation analysis, the results support the hypothesized associations between latent constructs and exercise behaviour in the related steps of the SDT process model. Autonomous motivation was found to act as a mediating variable in the relationship between psychological need satisfaction and exercise. We also found some of the paths to differ across age and gender, which could indicate that mechanisms in the SDT process model could vary (qualitatively) depending on subgroup, which calls for further exploration in future research.
